# Huperzine A and Huperzine B Production by Prothallus Cultures of *Huperzia selago* (L.) Bernh. ex Schrank et Mart

**DOI:** 10.3390/molecules25143262

**Published:** 2020-07-17

**Authors:** Wojciech J. Szypuła, Beata Wileńska, Aleksandra Misicka, Agnieszka Pietrosiuk

**Affiliations:** 1 Department of Pharmaceutical Biology and Medicinal Plants Biotechnology, Faculty of Pharmacy, Medical University of Warsaw, 1 Banacha St., 02-097 Warsaw, Poland; agnieszka.pietrosiuk@wum.edu.pl; 2 Faculty of Chemistry, University of Warsaw, 1 Pasteura St., 02-093 Warsaw, Poland; bwilenska@chem.uw.edu.pl (B.W.); misicka@chem.uw.edu.pl (A.M.); 3 Biological and Chemical Research Centre, 101 Żwirki i Wigury St., 02-097 Warsaw, Poland

**Keywords:** huperzine, *Huperzia selago*, prothallus, gametophyte, in vitro culture, secondary metabolites, Alzheimer’s disease

## Abstract

This is the first report of an efficient and effective procedure to optimize the biosynthesis of huperzine A (HupA) and huperzine B (HupB) in vitro from *Huperzia selago* gametophytes. Axenic tissue cultures were established using spores collected from the sporophytes growing in the wild. The prothalia were obtained after 7–18 months. Approximately 90 up to 100% of the gametophytes were viable and grew rapidly after each transfer on to a fresh medium every 3 months. The best biomass growth index for prothallus calculated on a fresh (FW) and dry weight (DW) basis, at 24 weeks of culture, was 2500% (FW) and 2200% (DW), respectively. The huperzine A content in the gametophytes was very high and ranged from 0.74 mg/g to 4.73 mg/g DW. The highest yield HupA biosynthesis at >4 mg/g DW was observed on W/S medium without growth regulators at 8 to 24 weeks of culture. The highest HupB content ranged from 0.10 mg/g to 0.52 mg/g DW and was obtained on the same medium. The results demonstrate the superiority of *H. selago* gametophyte cultures, with the level of HupA biosynthesis approximately 42% higher compared to sporophyte cultures and 35-fold higher than when the alkaloid was isolated from *H. serrata,* its current source for the pharmaceutical industry. Moreover, the biosynthesis of HupB was several-fold more efficient than in *H. selago* sporophytes growing in the wild. HPLC-HR-MS analyses of the extracts identified eight new alkaloids previously unreported in *H. selago*: deacetylfawcettine, fawcettimine, 16-hydroxyhuperzine B, deacetyllycoclavine, annopodine, lycopecurine, des-*N*-methylfastigiatine and flabelline.

## 1. Introduction

Huperzine A (HupA, selagine) and huperzine B (HupB) are two alkaloids which were first isolated from two Lycopodium species in the family Huperziaceae: *Huperzia serrata* and *Huperzia selago* (formerly known as *L. selago*) [1,2,3,4]. Pharmacological studies, in vitro, conducted in the search for novel drugs which would improve the symptoms of Alzheimer’s disease (AD), have demonstrated that both compounds are potent and reversible acetylcholinesterase (AChE) inhibitors which cross the blood-brain barrier [5,6]. The currently used treatments for AD rely on the use of AChE inhibitors because acetylcholine levels in the brain increase secondary to the inhibition of the enzyme which catalyzes its breakdown, and normal function of the cholinergic system in the brain is maintained [4,7,8]. Acetylcholine is a neurotransmitter which plays an extremely important role in memory, and increasing its levels at the synapses improves the acetylcholine transport and ameliorates cognitive disorders characteristic of AD. Animal and clinical studies have demonstrated that HupA is as effective in AD as the approved medicinal products, but it has a superior safety profile with fewer and less severe adverse effects [4,6,9]. Compared to the available AChE inhibitors, such as rivastigmine, donepezil and tacrine, HupA has high oral bioavailability, produces sustained AChE inhibition and is better transported across the blood-brain barrier [9]. Recently, more medicinal properties of HupA, other than AChE inhibition, have been discovered. HupA has been found to prevent apoptosis of neurons responsible for cognition and memory via reducing oxidative stress, stimulating expression of the neuronal growth factor (NGF) gene and NGF secretion and modifying *β*-amyloid transformation [6,7,10,11]. HupA promotes the non-amyloidogenic pathway of the *β*-amyloid precursor protein thereby reducing its accumulation in cells [6,7]. Importantly, HupA increases the activity of the antioxidant enzymes glutathione peroxidase, superoxide dismutase and catalase and improves cell survival. Studies suggest that pharmacological properties of HupA result from its effect on the genes coding for proteins of the Bcl-2 family, Bax and p53 [6,7,11]. The overexpression of the *BCL2* gene inhibits apoptosis, while the overexpression of *BAX* and *TP53* genes induces apoptosis. In an experimental rat model, after administration of HupA followed by apoptosis-promoting stressors, proapoptotic cytosolic alterations and decreased expression of *BCL2*, *BAX* and *TP53* genes were observed [6,7].

There are not many published studies on the pharmacological properties of huperzine B. HupB is a known potent and reversible AChE inhibitor with confirmed highly selective inhibition of AChE in the brain and less severe peripheral cholinergic side-effects [4]. According to Feng et al. [12], HupB (IC_50_ = 1930 nM) is a weaker inhibitor of AChE than HupA (IC_50_ = 72.4 nM). However, it has a greater therapeutic index and hence a more favorable safety profile [13]. It is a more potent agent than galantamine, but weaker than neostigmine, physostigmine and HupA [14]. It has a neuroprotective action and slows the progression of dementia [7]. It has also been demonstrated that HupB can protect neuron-like rat pheochromocytoma cells against damage caused by oxygen and glucose deprivation [14,15].

The discovery of the medicinal properties of HupA, especially as a candidate drug for neurodegenerative diseases, has attracted interest in Lycopods (club mosses) as a potential source of this alkaloid for the pharmaceutical industry. Nowadays, HupA is procured from *Huperzia serrata* native to Asia. The excessive exploitation of the species has reduced its primary range and depleted the natural resources [15,16]. This is what happened in Yunnan Province of China where *H. serrata* used to be a common plant and now has the status of an endangered species [15,16]. A long and complicated life cycle of Lycopods is also responsible. The complete cycle from the germination of spores and the gametophyte formation to the sporophyte maturation takes at least 15 years. The mature plants are only a few centimeters in height and the HupA content in sporophytes growing in the wild is low, ranging from 0.005 to 0.01% [16,17,18]. In the search for alternative sources of HupA, phytochemical screening has been carried out of approximately 50 of the 400 taxa of the Lycopodiaceae *sensu lato* family [5,6]. 

Huperzine A can be isolated from just a few species of the genera *Huperzia* and *Phlegmariurus*, which are members of the Huperziaceae *sensu stricto* family, e.g., *H. herteriana, H. ovatifolia, P. carinatus*, *P. mingcheensis*, *H. pinifolia*, and *H. selago* [5,15,16,17,19,20,21]. Lim et al. [22] found HupA in 11 of 17 investigated *Huperzia* species. Members of the genus *Lycopodium* L. and *Diphasiastrum* Holub (Lycopodiaceae *sensu stricto*)*,* commonly found over large areas in Europe, America and Asia, are rich in a variety of alkaloids, mainly lycopodine and its derivatives [23]. As confirmed by the published studies, only a few members of the Lycopodiaceae family can synthesize HupA and HupB, which is another factor limiting the procurement of the raw plant material for the pharmaceutical industry [23].

With the growing interest in therapeutic applications of HupA, the demand for club mosses as a source of this and other alkaloids which are acetylcholinesterase inhibitors is increasing rapidly and studies on in vitro cultures of Lycopods are gaining in importance. Larger-scale HupA and HupB procurement using tissue culture techniques requires highly efficient and fast plant tissue growth and alkaloid biosynthesis in vitro [23]. Earlier studies [17,19,24] proposed ways to enhance the production of alkaloids and plant tissue in a variety of tissue culture models. To date, sporophytes have been mainly used as the culture-initiating material.

**Figure 1:**
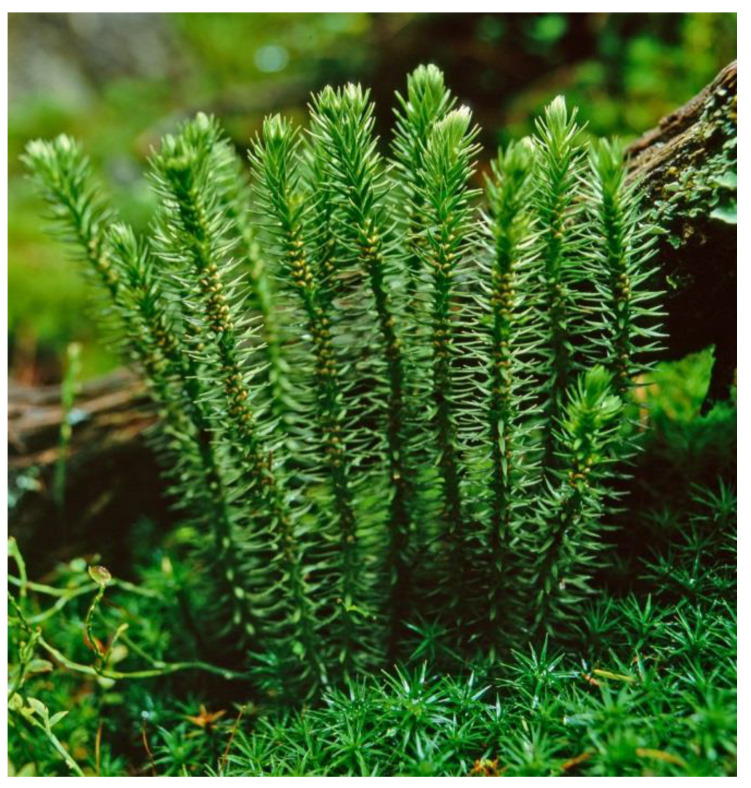
Fir clubmoss (*Huperzia selago*) sporophyte in its natural habitat in the Babia Góra National Park, Poland. Side view of the *H. selago* shoots presenting distribution of sporangia.

In the present study, we used the cultures *Huperzia selago* gametophytes for the biosynthesis of HupA and for the first time HupB. *H. selago* is the only European *Huperzia* species which contains HupA [5,20,23] (Figure 1). Importantly, *H. selago* is a rich source of HupA, since its sporophytes contain much more of the alkaloid than found in *Huperzia serrata* native to Asia [17,20,23]. The preliminary studies of HupA production in in vitro culture of *H. selago* gametophytes indicate that this is a more efficient method of obtaining secondary metabolites than sporophyte culture [17,23,25]. So far, there have been no studies on the biosynthesis of alkaloids or any other metabolites using in vitro culture of gametophytes (prothallus). Furthermore, there have been no studies to evaluate the effects of abiotic stresses such as light intensity and its spectral quality, sugar content, agar hydration, concentrations of mineral salts or growth regulators on the biosynthesis of HupA and other alkaloids found in the *Huperzia* species.

## 2. Results

### 2.1. Establishment of In Vitro Culture and Morphological Characteristics of Gametophytes

**Figure 2:**
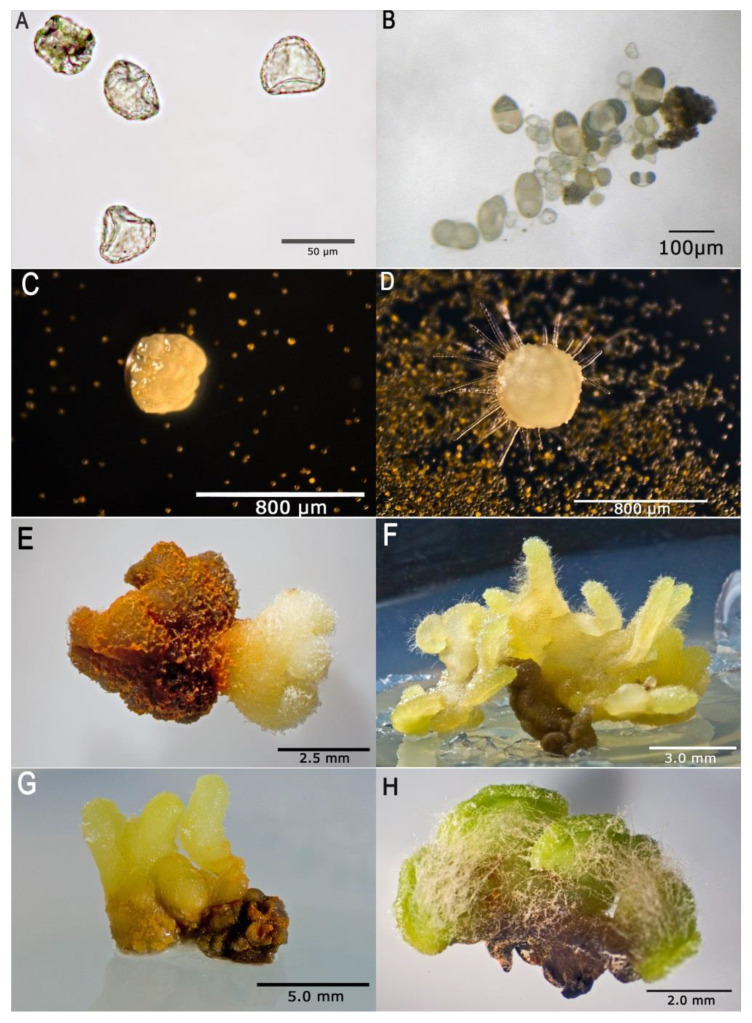
In vitro culture of spores and gametophytes of *Huperzia selago* for multiplication and an alkaloid production. (**A**) Spores on growth media for germination. (**B**) Germinating spores after 7 months of culture. (**C**) Young prothallus consisting of a few cells after 2–4 weeks. (**D**) Small spherical gametophytes after 3 months of culture. (**E**–**H**) Lateral view of different morphological types of the mature gametophyte and its late developmental stages.

The spores germinated, exclusively (0.1%) on the medium originally developed by Whittier and Storchova (W/S) and on Moore (MR) medium as modified by Freeberg and Wetmore [26,27], in the dark at 7 to 18 months of culture (Figure 2A,B). Importantly, only the spores collected in spring (April and May) germinated. At the stage of culture initiation, the spores were incubated for 24 h in 100 µM IAA and 50 µM kinetin and their walls were digested with enzymes. No spore germination and gametophyte growth were observed in repeated trials to achieve the germination of spores collected in other seasons and incubated on the other media tested. Similarly, no germination of spores which were scarified using the method of Freeberg and Wetmore [27] was observed at 24 months in culture, including the spores collected in April and May. Light was found to inhibit germination. When the same media and methods were used with which spore germination was achieved in cultures without light, no gametophytes developed during 24 months in culture in the light. With the media which allowed spore germination and further development of prothalli, the first cell division occurred inside the spore prior the wall rupture and produced two semicircular cells (Figure 2B). Their further division led to the development of the prothallus consisting of a few cells (Figure 2C). At 3 months in culture, the prothalli were 1–3 mm in diameter (Figure 2D). They matured after 6 to 12 months (Figure 2E–H) and did not show any signs of contamination. The mature prothalli were usually roughly square-shaped, long, narrow, axial structures with distinct dorsal and ventral surfaces (Figure 2). Most gametophytes had an hourglass- or disk-shaped cross section (Figure 2), but a smaller ventral side could cause narrower gametophytes to lose this shape (Figure 2). Generally, the mature gametophytes were dorsoventrally disk-shaped or cylindrical and branched. In culture, we also found other irregularly shaped gametophytes (carrot- or pincushion-shaped) but not filamentous-shaped gametophytes. 

**Figure 3:**
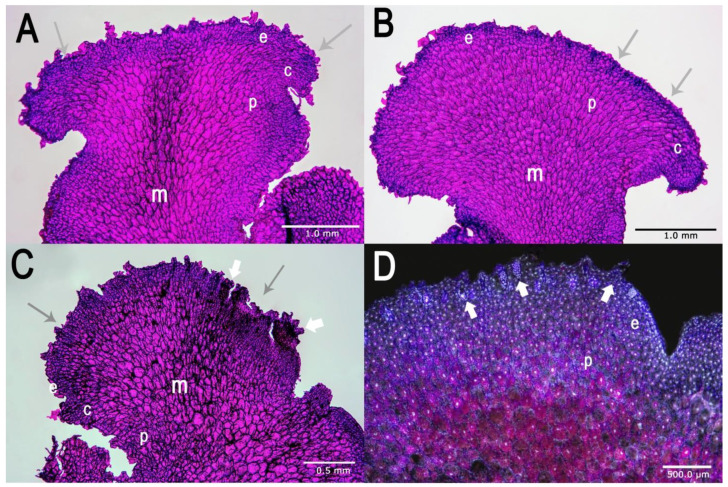
Light (**A**–**C**) and UV (**D**) micrographs of longitudinal sections of a different morphological type of *Huperzia selago* gametophytes. Picture shows an apical region of gametophytes with meristematic zones (arrows). Along the apical regions of some mature prothalli, clusters of gametangia were found (**C**,**D**, small white arrows); e-epidermis, c-cortex, p-palisade cells, m-central parenchymatous core.

**Figure 4:**
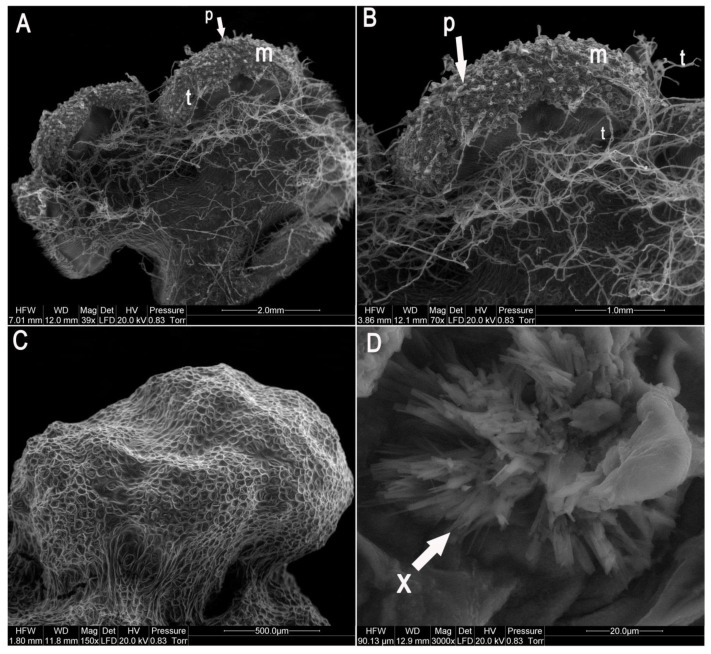
Development of the mature phase and adult gametophytes using SEM microscope. (**A**) Dorsal and ventral view of an adult, heart-shaped gametophyte. (**B**) Magnification ventral view of the same gametophyte. Detail of the notch showing meristematic cells with meristematic zone (m) and embedded in the epidermis antheridia with numerous sterile paraphyses (p). In most of the gametophytes numerous trichomes covered the ventral surface (t), but some prothallus (**C**) did not develop gametangia and their ventral surface was devoid of trichomes. (**D**) An orange-colored crystallized secretion at the base of the ventral surface of prothallus (X).

The mature gametophytes were on average 4–10 mm long (Figure 2, Figure 3 and Figure 4). Along the apical regions of some mature prothalli, clusters of gametangia were found (Figure 3D) and embedded in the epidermis antheridia with numerous sterile paraphyses (Figure 2F,H, Figure 3, Figure 4A,B). In most of the prothalli, numerous trichomes covered the ventral surface (Figure 3 and Figure 4A,B), but some prothalli did not develop gametangia and their ventral surface was devoid of trichomes (Figure 4C).

The vegetative part of the prothallus consisted of three distinct layers: the epidermis with heavily cutinized outer walls and trichomes, the cortex made up of several rows of ovoid cells and the palisade layer and the central parenchymatous core (Figure 3). The most important information on cultured gametophytes of *Huperzia selago* is summarized in Figure 2, Figure 3 and Figure 4.

When cultures were maintained, an orange-colored secretion was observed which usually collected at the base of the ventral surface (Figure 2E). The secretion diffused into the medium changing its color. Scanning electron microscopy of the secretion found that it crystallized (Figure 4D). For chemical identification, the crystals were separated from the tissue, dissolved in methanol to prepare 1 mg/mL concentration and analyzed using HPLC-DAD (High Performance Liquid Chromatography with Diode Array Detector) to identify alkaloids. The chromatograms showed about 14–15 peaks, of which two at 10.4 min and 10.9 min corresponded to HupA and HupB, respectively.

### 2.2. Effects of Media and Growth Regulators on Cell and Biomass Growth

**Figure 5:**
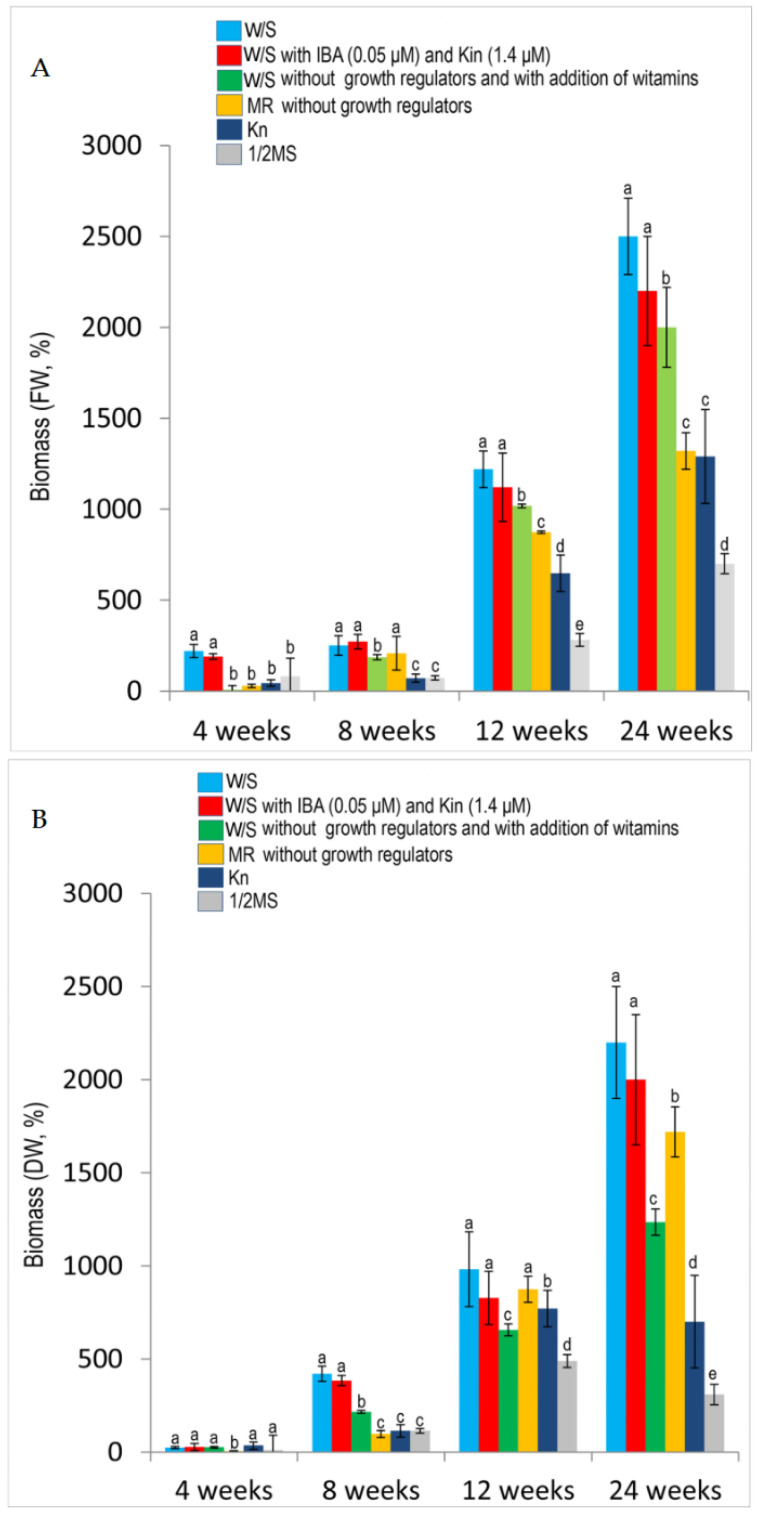
Effect of media and its composition on fresh (FW) (**A**) and dry (DW) (**B**) biomass increase in gametophytes culture. Data are expressed as mean ± SD of at least three independent experiments. Significantly different values are denoted by different lowercase letters (*p* < 0.05). The statistical evaluation of the effect of media and its composition on biomass growth was evaluated in individual weeks (successively in weeks 4, 8, 12 and 24) but not between weeks.

The media had different effects on the growth and viability of gametophytes. The media prepared according to the method of Whittier and Storchova (W/S) [26] without growth regulators or supplemented with indole-3-butyric acid (IBA) (0.005 µM) and kinetin (Kin) (1.4 µM) or such media without growth regulators but supplemented with vitamins proved the most effective. Good results were also achieved on MR medium [27]. In consecutive passages, cultures on these media yielded from 90 to 100% of viable, rapidly growing gametophytes. When Murashige and Skoog (½ MS) medium with modifications earlier used in the cultures of *H. selago* sporophytes was used [17], gradual slowing of the growth and development in the prothalli was observed. Data concerning the growth of gametophyte biomass on the media tested are summarized in Figure 5. The best prothallus biomass growth rate calculated on a fresh (FW) and dry weight (DW) basis at 24 weeks of culture was observed on the W/S medium without growth regulators and it was as high as 2500% (FW) and 2200% (DW). The mean biomass growth rate calculated for the other W/S was 2200% (FW) and 2000% (DW) for the medium with IBA (0.05 µM) and Kin (1.4 µM) and 2000% (FW) and 1235% (DW) for the medium without growth regulators and with addition of vitamins (Figure 5). Good results were observed with MR medium [27], when the growth rates were 1320% (FW) and 1720% (DW). Poor gametophyte growth on Kn [28] medium and ½ MS [17,29] medium with reduced mineral salt content was reflected in the biomass growth rates at 24 weeks, 1290% (FW) and 700% (DW) and 700% (FW) and 309% (DW), respectively, the lowest biomass growth rates observed in the present study. Variations among the mean biomass growth rates observed with some of the tested media were significantly greater than expected due to chance (Kruskal-Wallis one-way analysis of variance by ranks, *p* < 0.05). Details are presented in Figure 5.

### 2.3. Characterization of Huperzia Selago Alkaloids from Gametophytes Using HPLC-DAD and Mass Spectrometry

**Figure 6:**
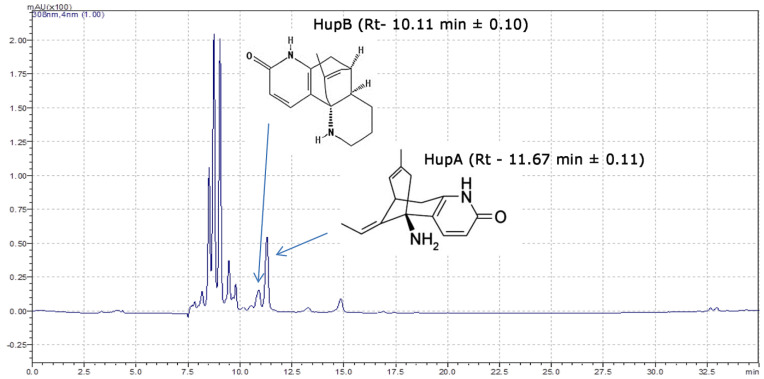
Sample chromatogram of *H. selago* extract (gametophytes from culture).

To determine the content of HupA and HupB and other alkaloids in *H. selago* gametophytes, dried alkaloid extracts were prepared and analyzed using HPLC-DAD and LCMS-IT-TOF. The extraction of HupA and HupB was achieved by following an established protocol [20]. The peaks corresponding to HupA and HupB were characterized by appropriate retention times, selectivity and resolution factors. The mean retention times for the HupA peak and the HupB peak were 11.67 min ± 0.11 and 10.11 min ± 0.10, respectively (Figure 6). The mean retention factor (k) for the HupA peak was 11.666 ± 0.143 min and remained within the recommended range of 0.5 < k < 20 [30]. The UV spectrum of standard HupA and HupB ranged from 190 to 800 nm and showed the absorption maxima at 231.8 and 307.7 nm for HupA and at 222.9 and 306.8 nm for HupB. In the high resolution mass spectrum of standard HupA, ions with *m*/*z* 234.1486 (difference between theoretical and measured *m*/*z* of HupA is 2.4 ppm) indicate the protonated ions [M + H]^+^ of HupA. For the HupB, ions with *m*/*z* 257.1643 (difference between theoretical and measured *m*/*z* of HupB is 1.9 ppm) indicate the protonated ions [M + H]^+^ of HupB (Table 1). 

In total, using high resolution mass spectrometry and Formula Predictor software (Shimadzu, Kyoto, Japan), 14 compounds were identified based on the comparison of their theoretical mass and molecular formula (based on the literature data) with measured mass and proposed molecular formula (Table 1). Most of these were known compounds earlier isolated from sporophytes of Lycopodiaceae *sensu lato* but never from the prothallus, which confirmed that most, if not all, of these identifications were valid.

### 2.4. Effects of Different Media and Their Composition on Huperzine A and Huperzine B Production

**Figure 7:**
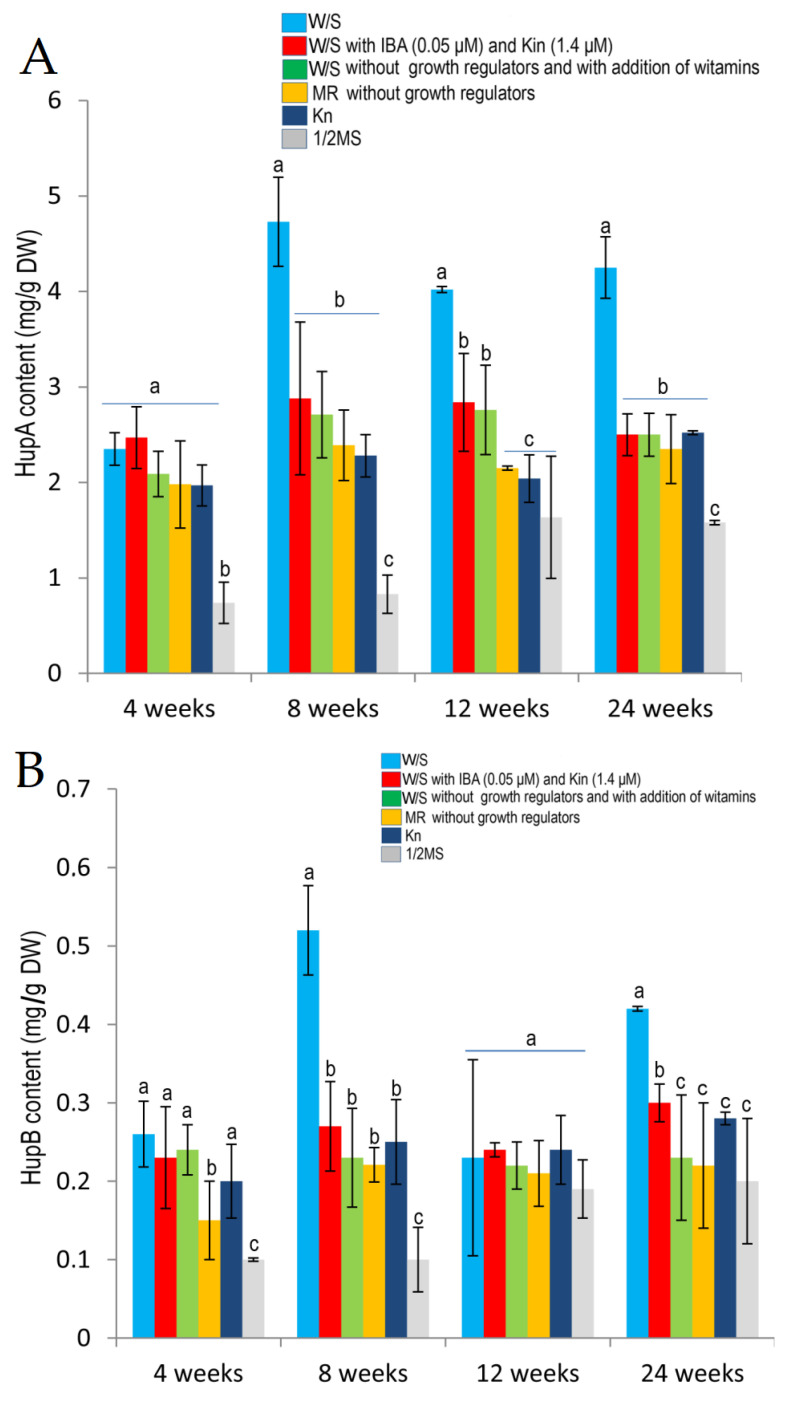
Total content of HupA (**A**) and HupB (**B**) in prothallus from in vitro cultures after 4, 8, 12 and 24 weeks on different media. The results are means of three replicates (*n* = 3) ± SD and are from three independent experiments. Significantly different values are denoted by different lowercase letters (*p* < 0.05). The statistical evaluation of the effect of media and its composition on an alkaloids synthesis was evaluated in individual weeks (successively in weeks 4, 8, 12 and 24) but not between weeks.

The content of huperzine A in the gametophytes from in vitro culture was very high and ranged from 0.74 mg/g to 4.73 mg/g dry weight (DW) (Figure 6). The highest HupA content, 4.73 mg/g DW, was found in the gametophytes at 8 weeks of culture on W/S medium without growth regulators. With the same medium, the highest stable increase in the biosynthesis of HupA during the entire culture period was observed: 2.35 mg/g DW at 4 weeks to over 4.0 mg/g DW in the subsequent weeks (Figure 7). Importantly, from 8 to 24 weeks of culture this increase in the HupA content with W/S medium was statistically significant in comparison to cultures using the other media. The mean HupA content in gametophytes during 24 weeks of culture on W/S medium was 3.84 ± 0.27 mg/g DW. 

During the 24 weeks of the experiment, a high HupA content of gametophytes, which on average ranged from to 2.20 ± 0.11 to 2.67 ± 0.25 mg/g DW, was observed on the other media according to Whittier and Storchova (W/S) [26], Moor (MR) medium as modified by Freeberg and Wetmore [27] and Knudson medium (Kn) [28] (Figure 7). The lowest HupA content was found in the gametophytes cultured on ½ MS medium [17,29]. It ranged from 0.74 mg/g DW to 1.635 mg/g DW at 12 weeks of culture (mean content: 1.20 ± 0.26 mg/g DW).

The content of huperzine B in the gametophytes from in vitro culture ranged from 0.10 mg/g to 0.52 mg/g DW (Figure 7). As with HupA, the highest content of HupB in gametophytes was found at 8 weeks of culture on W/S medium without growth regulators. The mean HupB content of gametophytes during 24 weeks of culture was 0.36 ± 0.05 mg/g DW.

With the other media, a comparable level of HupB biosynthesis was achieved for the entire length of culture, on average from 0.24 ± 0.02 to 0.26 ± 0.03 HupB per 1 g DW. (Figure 7). The lowest Hup B content was found in the gametophytes cultured on ½ MS. It ranged from 0.1 to 0.2 mg/g at 24 weeks of culture (mean content for the entire culture length: 0.15 ± 0.03 mg/g DW).

## 3. Discussion and Conclusions

This paper presents a protocol for a fast and effective in vitro axenic culture of *Huperzia selago* (L.) Bernh. ex Schrank et Mart. (=*Lycopodium selago* L., fir club moss, Huperziaceae Rothm) gametophytes (prothallus), which is the best available and rich source of huperzine A (HupA, selagine) and other alkaloids. These compounds have a considerable therapeutic potential and are being extensively investigated for uses as treatments for a number of diseases [5,6,11,31,32]. The major therapeutic interest described for HupA is as treatment for acetylcholine-deficit dementia, including Alzheimer’s disease [6]. Our previous results [31,32] demonstrated for the first time that alkaloids from *H. selago* possess antioxidative properties and scavenge free radicals as well as prevent lipid and protein oxidation, presenting the desired mechanism of action in neurodegenerative disorders. These alkaloids might be a promising source of active constituents of novel treatments for Alzheimer’s and Parkinson’s diseases [6,8]. Importantly, *H. selago* is the only European and North American species which contains HupA [17,23]. Studies have shown that the plant is a rich source of HupA, much more abundant than the Chinese club moss *Huperzia serrata*. 

To date, studies on in vitro cultures have been conducted with a limited number of club moss species. Moreover, in the literature, there are only a few protocols for the propagation of sporophytes of HupA-containing club mosses and no effective protocols for the prothallus culture [23]. Up to now, club moss gametophytes from natural populations have been only infrequently described and in very rare cases they were obtained in vitro, but they have not been effectively used for the biosynthesis of secondary metabolites [23]. The first studies on the biosynthesis of HupA were conducted by Szypuła et al. [17,20,25] and Ma and Gang [24]. Szypuła et al. [17] presented a protocol for the establishment and maintenance of in vitro culture of *Huperzia selago* sporophytes from shoot explants (Figure 1). Using the method of indirect somatic embryogenesis, the authors obtained somatic embryos which after 6 months in culture produced sporophytes with the highest HupA content reported to date in Lycopods, as much as 3.33 mg/g DW. Other research groups established in vitro cultures of *H. serrata* and a few other closely related HupA-producing species [24]. The obtained *Phlegmariurus squarrosuus* tissues were found to produce satisfying amounts of HupA (from 0.312 to 0.675 mg/g DW in gametophytes and from 0.434 to 0.675 mg/g DW in sporophytes). Ishiuchi et al. [19] evaluated the alkaloid content in samples of 11 *Huperzia* species cultivated in greenhouses and in vitro. *H. pinifolia* in vitro culture was established and the biomass was assessed for alkaloid content (the maximum HupA content was 0.286 mg/g DW). In vitro cultures of the remaining *Huperzia* species were also initiated but the growth of plant tissue was not satisfactory. Of the 11 species evaluated, HupA was produced by the sporophytes of 10 species. The highest HupA content was found in *H. pinifolia* (1.765 mg/g DW), followed by *H. nummulariifolia* (1.69 mg/g DW). These results demonstrate that the maximum HupA content in the evaluated species was approximately 26 times [17] and 13 times [19] higher than the mean HupA level in *H. serrata* whole plant and 10 times higher than the maximum HupA content in *H. serrata* sporophytes characterized by the most biosynthesis of HupA. 

In nature, the gametophytes of different club moss species are deeply subterranean (gametophyte development may take place even 20 cm below the surface of the ground), holosaprophytic and mycotrophic [33]. Usually, they are tubercular, conical in shape and devoid of chlorophyll [23,27,33]. In the genus *Lycopodiella* Holub, the prothallia possess chloroplasts [23]. The average prothallus is 8 mm long and 4 mm wide, but some may be as long as 20 mm and 5 mm wide [34]. Aggregates of antheridia and archegonia form the upper lobed crown region. Generally, the gametophytes are monoecious (bisexual), but some possess antheridia only [35]. The vegetative part of the prothallus is made of four layers: the outermost epidermis with heavily cutinized walls and trichomes which are epidermal outgrowths, the cortical layer beneath the epidermis, composed of up to eight rows of ovoid cells with a high intracellular content of fungal hyphae, and the palisade layer, which consists of one row of strongly elongated cells at right angles to the prothallus surface and serves as storage tissue. The parenchymatous central core is the innermost layer [35]. In nature, the germinating gametophyte does not live independently after it has divided into a few cells [27]. Association with fungi is obligatory for further gametophyte development as the mycelium of the fungus developing in the prothallus is in direct contact with mycelium in the soil and soluble nutrients found in the humus pass into the cells of the prothallus. In nature, the spore germination takes 2–3 years and the gametophyte maturation will take another 12–15 years [33]. Freeberg and Wetmore [27] and Freeberg [36,37] described the gametophytes of *Lycopodium* grown in vitro. Other partially successful cultures involved the gametophytes of *Lycopodium obscurum* [38], *L. digitatum* [39], *L. lucidulum* [40] and *H. selago* [26]. In the present study an efficient in vitro *H. selago* prothallus induction system was established on different Moore (MR) media as modified by Freeberg and Wetmore [27] and Whittier and Storchova [26], Knudson [28], and Murashige and Skoog (MS) [29] with or without growth regulators, supplemented with 2.5 or 5 g/L glucose and different concentrations (0.05–1.4 μM) of growth regulators, i.e., auxins (IBA) and cytokinins (kinetin), in the dark. Gametophyte cultures were initiated using spores obtained from populations growing in the wild. Their harvesting does not deplete the natural populations and is safe for club moss species threatened with extinction or for small local populations. Importantly, spore disinfection according the protocol developed specifically for the present study allowed tissue cultures free from bacterial and fungal contamination to be established and maintained. Achieving axenic cultures of the sporophytes of *H. selago* and other club mosses is the most challenging step [17,23,25]. Club mosses are mycorrhizal plants and their aboveground tissues are colonized by endophytic bacteria and fungi. They are found in gametophytes and sporophytes—in the mesophyll, air spaces between the cells of the cortex and in the cells of vascular tissues [17,23,37]. High mycorrhizal and endophytic species diversity encountered with club mosses considerably limits the use of sporophyte explants (shoot fragments or propagules) to initiate tissue cultures. Sporophytes used for that purposes require complicated and time-consuming disinfection procedures involving surface disinfection and intratissue antibiotic treatment, which, however, destroy some of endophytic species only [17,20,23]. According to the published literature, approximately 10% of explants in established cultures of long duration reveal the signs of contamination [17,19]. When commercial bulk-scale long-term tissue cultures are considered, the contamination issue would considerably impact the use of sporophytes for alkaloid production. So far, there has been only one method of sporophyte regeneration, i.e., somatic embryogenesis, which allows axenic Lycopod cultures to be established and maintained. To date, somatic embryogenesis has been described for just two Lycopodiaceas species, *Lycopodiella inundata* [41] and *Huperzia selago* [17], and of these, only *H. selago* can synthesize HupA. The use of somatic embryogenesis is, however, difficult, time-consuming and not very effective, although the sporophytes regenerated from somatic embryos contained 3.33 mg/g DW of HupA, the highest so far obtained amount of HupA [17]. In the present study, we wanted to determine the effects of culture conditions on the increase of gametophyte tissue mass and alkaloid synthesis levels. Low nutrient culture media proved to be suitable for successful gametophyte culture. Those were the media according to Whittier and Storchova [26], in fact, modifications of Moore medium which was used by Freeberg and Wetmore [27] and Freeberg [36] in the first reported in vitro cultures of club mosses. The choice of growth regulators was guided by earlier published reports of in vitro cultures of sporophytes of different club moss species, including the paper by Atmane et al. [41] on somatic embryogenesis in *Lycopodiella inundata*. In the sporophyte cultures of *H. selago*, supplementation of MS medium with IBA and kinetin was usually phytotoxic for fragments of *H. selago* shoots obtained from sporophytes growing in the wild [17]. However, in the cultures of gametophytes, supplementation of W/S medium with 0.05 μM IBA and 1.4 μM kinetin stimulated prothallus maturation and development of the gametangia. Similarly, Atmane et al. [41] observed that addition of IBA (0.05 μM) and kinetin (1.4 μM) to MS medium stimulated callus proliferation in the culture of *Lycopodiella inundata* shoots. The results of the few studies on tissue cultures of club mosses demonstrate that, apart from the endogenous properties of the explants, the success of the culture largely depends on the suitable composition of the basal medium, the growth regulators used and the source of nitrogen and the form in which it is presented. Nitrogen in the form of ammonium nitrate has long been known to determine the direction of explant morphogenesis [42,43,44]. It stimulates somatic embryogenesis, but the mechanism of this phenomenon remains unclear. It is generally assumed that substances supplying reduced nitrogen can stimulate protein synthesis and in this way impact morphogenesis [43,44,45]. The published literature suggests that such components of a culture medium as mineral salts or vitamins play a limited role [17,42,43,44,45]. As demonstrated in the present study, the use of MS medium rich in reduced nitrogen produced the least increase in biomass growth. Similarly, MS medium proved not suitable for sporophyte cultures [25]. The fastest gametophyte biomass growth was observed on W/S media without growth regulators. The growth rate of *H. selago* gametophytes calculated on a fresh and dry weight basis was nearly 15-fold higher than the growth rate of sporophytes obtained from shoot fragments at the same length of culture (6 months) and nearly twice as high as the growth rate in the cultures of sporophytes obtained from propagules via somatic embryogenesis [17,23,25].

To date, no procedures have been developed for the synthesis and obtaining alkaloids in tissue cultures of club mosses on a commercial scale, while published studies show that gametophyte culture may be a simpler, more efficient and less expensive method of alkaloid biosynthesis than sporophyte culture.

The present study shows that the tissues of *H. selago* gametophytes are capable of synthesizing alkaloids, including HupA and HupB. There are no earlier studies of secondary metabolites produced by *H. selago* gametophytes, although there has been one trial using in vitro culture of *Phlegmariurus squarrosuus* metabolites for the biosynthesis of HupA, with a HupA yield at 6 and 12 months of 0.312 mg/g to 0.675 mg/g DW. In the present study, the biosynthesis of HupA and HupB by *H. selago* gametophytes was maintained throughout the whole experiment (6 months). HPLC-DAD and LC-MS analyses demonstrated that the qualitative composition of major alkaloids found in the gametophytes was similar to the alkaloid complex in the sporophyte tissues [17,20]. Importantly, the amount of HupA in the cultured gametophytes was on average nearly double the amount of HupA in the investigated sporophytes of various club moss species procured from their natural habitats. As much as up to 4.73 mg (0.47%) of HupA per 1 g DW was found in the gametophytes from in vitro cultures. Before that, the highest HupA content was found in the sporophytes cultured from somatic embryos, i.e., 3.33 mg/g DW (0.33%) [17]. The results demonstrate that by using gametophytes it is possible to increase HupA biosynthesis by approximately 42% compared to sporophyte culture. Considering that the mean content of Hup A in the sporophytes of *H. serrata* ranges from 0.0047 to 0.025% [6], with *H. selago* gametophytes the yield of the alkaloid is even 35-fold higher than in *H. serrata.*

There are not many published studies on the HupB content in plant material or papers comparing the proportions of HupA and HupB in plants. HupB was earlier found in a limited number of club moss species, but the exact levels have been determined only recently [5,46]. According to Xu et al. [46], *H. selago* sporophytes from populations in Iceland contained approximately 0.006 to 0.2 mg HupB in 1 g of plant material. The exact relationship between HupA and HupB contents in investigated *H. selago* plant material is not known, but sporophytes from the Icelandic populations contain several hundred times less HupB than HupA in the same plant material [46]. The content of HupB in the *H. selago* gametophytes we obtained in vitro ranged from approximately 0.10 mg/g DW to 0.52 mg/g DW, which reflects very efficient biosynthesis of this alkaloid compared to the sporophytes of the same species.

According to the published literature, at least 13 alkaloids are present in *H. selago* tissues, including acrifoline, 12-epilycodoline (pseudoselagine), lycodoline, licopodine, selagoline, serratidinina, 6*β*-hydroxyhuperzine A, *α*- and *β*-obscurine, huperzine B, serratine and lucidoline [3,47,48,49,50,51,52]. In the present study, HPLC-HR-MS analyses of the alkaloid extracts from the gametophytes demonstrated a number of alkaloids which are either not present in *H. selago* sporophytes or their presence has not been confirmed. These are deacetylfawcettine, fawcettimine, 16-hydroxyhuperzine B, deacetyllycoclavine, annopodine, lycopecurine, des-*N*-methylfastigiatine and flabelline. The presence of annopodine is an interesting finding since until now it has been considered as characteristic of the *Lycopodium sensu stricto* clade and thought to be limited to the *Lycopodium annotinum* species [5,23], while according to some authors, the group of taxa which includes *L annotinum* constitutes a distinct genus of *Spinulum* Haines. Their opponents point out the absence of significant differences in analyses using molecular markers which would support distinguishing of a new genus. Analyses using molecular data demonstrate that the genus *Huperzia* is the oldest club moss group, while *H. selago* is the oldest representative of the genus, taxonomically remote from the *Lycopodium* clade and with a different evolutionary lineage [23]. The presence of annopodine in the *H. selago* gametophytes would be an instance of chemical convergence rather than of chemotaxonomic relationship, although the biosynthesis of all club moss alkaloids follows a similar pathway with lysine acting as a precursor. Of the alkaloids found in *H. selago* sporophytes, in addition to HupA and HupB, the gametophytes contain four: 6*β*-hydroxyhuperzine A, serratinidine, lycopodine and selagoline. Interestingly, these alkaloids are not among approximately 90 alkaloids so far isolated from *H. serrata* [5,15,16,23].

The results of the present study and earlier findings reported by Atmane et al. [41], Szypuła et al. [17,23], Ma and Gang [24] and Ishiuchi et al. [19] indicate that in tissue cultures the life cycle of club mosses is dysregulated to a greater or lesser extent since the explants are not controlled by the regulatory mechanisms of the whole plant and may be subjected to stresses such as excess or deficiency of sugars or growth regulators in the medium or its hydration status. In practice, when studying the biosynthesis of pharmacologically active compounds, this specific, altered and manipulated life cycle may be utilized to study the factors controlling the periodic developmental stage-related variations in the biosynthesis of selected alkaloids in sporophytes or gametophytes. In the future, exact knowledge of the life cycle alterations under experimental conditions as well as a clear understanding of the typical life cycle and its numerous modifications in vivo could be the key to the choice of those tissues and organs of club mosses which are best suited to isolating alkaloids. 

## 4. Materials and Methods

### 4.1. Reagents and Standards

The following reagents and standard substances were used in the tests: Acetonitrile HPLC grade, Methanol HPLC grade Chromasolv^®^, Sodium hexafluorophosphate (NaPF_6_) and Ammonium acetate. Cellulase and Pectinase were purchased from Sigma-Aldrich Chemie GmbH (Steinheim, Germany). HPLC grade water was purchased from Merck KGaA (Darmstad, Germany). LC-MS H_2_O (18 Ω) grade was purchased from Millipore Merck KGaA (Darmstadt, Germany). LC-MS acetonitrile grade was purchased from Merck. Chloroform, diethyl ether, sodium chloride, ethyl alcohol, formaldehyde 37% and glacial acetic were purchased from POCH (Gliwice, Poland). Bacto™ Agar was purchased from Becton, Dickinson & Company, (Franklin Lakes, New Jersey, USA). IAA, IBA and kinetin were purchased from Grand Island Biological Company (New York, USA). Plant Preservative Mixture (PPM) was purchased from Plant Cell Technology (Washington, D.C., USA). Toluidine Blue was purchased from Across Organics (Geel, Belgium). Periodic acid and Schiff’s reagent for microscopy was purchased from Merck KGaA. Huperzine A and Huperzine B standards were purchased from ChromaDex Inc. (Laguna Hills, CA, USA).

### 4.2. Plant Material and Establishment of In Vitro Culture 

*Huperzia selago* gametophyte in vitro cultures were initiated using spores obtained from shoot fragments of sporangia-bearing sporophytes procured from populations in the Babia Góra National Park (49°34′24″ N 19°31′46″ E), The Beskids, Poland (Figure 1). The plants were harvested at 30-day intervals, from April through November 2013.

Voucher specimens, including sporophytes with sporangia, were taken and deposited in the Herbarium at the Faculty of Biology, University of Warsaw (WA) (Nos 0000074900). The plant was identified by Dr. W. Szypuła, according to the literature data [23,53].

The plants were sorted and removed to the laboratory. They were cleansed of dirt and debris, transferred to Petri dishes lined with filter paper and incubated at room temperature for a few days to achieve spore release. The spores were passed through a 0.5 mm sieve and afterwards stored in the dark at room temperature for up to three weeks and then used to initiate gametophyte cultures.

Prior to establishing in vitro cultures, the spores were disinfected using our own method of surface disinfection developed for in vitro culture of *H. selago* sporophytes [25], which consisted of soaking the spores in 70% ethanol (*v*/*v*) for 1 min, followed by sodium hypochlorite (ACE, Procter & Gamble Cincinnati, Ohio, USA) with H_2_O (1:5 *v*/*v—*which is 0.8% sodium hypochlorite solution) for 10 min and next 7% *v*/*v* H_2_O_2_ for 5 min.

In nature, the germination of *H. selago* spores takes approximately 3 to 5 years and that is why the next mandatory step involved mechanical or chemical scarification treatment of the spores to overcome dormancy and speed up germination. The method described by Freeberg and Wetmore [27] was used or the spores were soaked for 24 h at 20 °C in the dark in the solution of growth regulators (100 µM IAA and 50 µM kinetin). Moreover, the spores were separated from the growth regulator solution using fluted filter papers and gravity filtration and again soaked in water with 1% (*v*/*v*) cellulase and 0.5% (*v*/*v*) pectinase for 6 h at 24 °C. 

After disinfection and the stage of dormancy break, the spores (500 mg) were suspended in 20 mL of sterile distilled water and next 2 mL of the suspension was transferred to growth media for germination. The germination of each portion of spores, collected in different seasons, was evaluated on the following tested media: W/S [26] without growth regulators, W/S with IBA (0.05 µM) and kinetin (Kin) (1.4 µM), W/S without growth regulators and with addition of vitamins, and on Moore medium (MR) [27] without growth regulators. In addition, the media according to Knudson (Kn) [28] and Murashige and Skoog (½ MS) [29] with half strength mineral salt content, both without growth regulators, were tested. The W/S and MR media were supplemented with glucose (2.5 g/L) and agar (1.1%). The ½ MS medium was supplemented with 10 g/L saccharose and 0.8% agar. All media were adjusted to pH 5.9 prior to autoclaving. Plant Preservative Mixture™ (PPM™; Plant Cell Technology, Washington, USA) 2 mL/L was added to the media to prevent microbial contamination during the stage of spore germination, which lasted several months.

The control cultures were initiated from spores collected in the same period of time (from April through November) but which were not scarified mechanically or treated with growth regulators or the enzymes cellulase and pectinase. Before sowing they were soaked in water without growth regulators for 24 h at 20 °C in the dark.

The spores, on all the media listed above, were incubated in a phytotron at 18 ± 1 °C (day) and 16 ± 1 °C (night) with light at 100 μM/m^2^/s and 14 h photoperiod or without light, at the same temperature. 

After the germination of spores, 5 g/L glucose and 1.1% agar were added to the MR and W/S media. The gametophytes were transferred on to a fresh medium every 3 months. The prothallia were incubated in a phytotron at 18 ± 1 °C (day) and 16 ± 1 °C (night) in the dark.

### 4.3. Morphological Analysis of Huperzia Selago Prothallus Cultures

To observe all stages of gametophyte development, a Discovery V12 (Zeiss) stereo light and fluorescent microscope with Apochromat S 0.63× and PlanApo S 1.5× lenses equipped with a Nikon D90 camera was used. Scanning electron microscopy (SEM) was used for morphological observation of the prothallus. Various prothallus specimens were directly inserted inside an environmental scanning electron microscope (ESEM) QUANTA 200 FEI (FEI Europe B.V., Eindhoven, Netherlands), suitable for observation of biological material, without prior fixation. Secondary electron images (SE) were taken at 20 kV. Photographs were taken at different magnifications. At 4, 8, 12 and 24 weeks in culture, the index of gametophyte fresh and dry biomass growth (WP) was calculated for 30 prothalli and the calculation was repeated in triplicate. The WP value was calculated according to Street and Henshaw [54] where WP = final plant mass (g) − initial plant mass (g)/initial plant mass (g) × 100. The final results are presented as the mean WP value for the entire harvest period.

### 4.4. Histological Analysis

Analyses were carried out on juvenile and mature prothalli cultured on W/S medium without growth regulators. Their stage of maturity was identified by morphological features described by Whittier and Storchova [26]. For each analysis, at least 10 prothalli were used. Photographs shown in this paper are representative examples (Figure 3). The samples (prothallus) were vacuum-infiltrated with FAE fixative with formaldehyde 37%:100% glacial acetic acid:ethanol 50%, 6.5:3.5:100 mL (*v*/*v*/*v*) for 72 h at room temperature [39]. Fixed tissue was dehydrated in a series of ethanol concentrations: 40, 50, 70, 90, 100% (*v*/*v*) (30 min each bath). After dehydration and wax (Paraplast Plus, McCormic Scientific) embedding 5–10 µm specimens were cut. Sections were stained with PAS (periodic acid-Schiff) or double stained with PAS and NBB (Naphtol Blue Black). PAS specifically stained polysaccharides red, and NBB stained soluble and reserve proteins bluish-black. Observations and slides showing important features were made with a light Zeiss Axio Lab A1 microscope equipped with a Canon EOS 1100D camera. Additionally, some microscopic analyses were based on light blue autofluorescence of mature archegonia and red autofluorescence of chlorophyll with a standard filter set (excitation/emission 358/461 nm) with the same microscope.

### 4.5. Alkaloid Extraction and Determination

#### 4.5.1. Preparation of Extracts and Their Purification

An alkaloid fraction was obtained using a conventional procedure from methanol extract, as previously reported by Szypuła et al. [20]. Each sample of powdered plant sample of prothallus (0.25 g) was extracted with methanol of analytical purity grade (5 mL) for 30 min, repeated three times until the eluate was negative to Dragendorff’s reagent. Then, the combined extracts were filtered. The extraction was carried out in a RK 100H ultrasonic cleaning bath (Bandelin Sonorex, Berlin, Germany), with a mean operating frequency of 35 kHz and the power adjusted to 160 W, at 40 °C. The combined filtrates were evaporated under reduced pressure. Next, the dry residue was dissolved in 10 mL of 2.5% hydrochloric acid and purified by shaking twice with chloroform and then with ethylic ether. The water phase was rendered basic with 25% ammonia solution (pH 9), salted out with sodium chloride and then exhaustively extracted with chloroform. In the last stage, the combined chloroform extracts were evaporated to dryness. The dry residue was dissolved in 1 mL of methanol (HPLC purity grade) and used in the HPLC-DAD assay.

#### 4.5.2. Qualitative and Quantitative HPLC-DAD Analyses of HupA and HupB

Qualitative and quantitative HPLC–DAD analyses of HupA and HupB were performed on a Shimadzu system (Kyoto, Japan) consisting of a UV-VIS and SPD-10A DAD 340S detector, LC-10AD pump and LC solution software. A Hypersil GOLD column, C_18_ 250 × 4.6 mm, and a Hypersil GOLD precolumn, 5UM 10 × 4 mm, were used. The mobile phase consisted of (A) water with the addition of 30 mM NaPF_6_ and (B) acetonitrile. The following gradient was applied: 0–5 min 0→25% B, 5–20 min 25→45% B, 20–30 min 45→80% B [20]. Column temperature: 24 ± 1 °C, flow rate of the mobile phase: 1.0 mL/min, analysis duration: 30 min, detection at wave lengths λ = 210, 230, 260, 308 nm. The HPLC–DAD analyses of HupA and HupB used in this work was previously validated [20].

#### 4.5.3. LC-MS Analysis of HupA and HupB

A Shimadzu Prominence high-performance liquid chromatograph (HPLC) was used coupled with a LCMS-IT-TOF mass spectrometer (Shimadzu), equipped with an ion trap (IT), a time-of-flight (TOF) detector and an electrospray ionization (ESI) source. Mass spectra were recorded in the positive ion mode using LCMS solution software (Shimadzu).

Conditions for HPLC separation and detection of alkaloids were as follows: column Kinetex C_18_, 2.6 µm, 2.1 × 100 mm, Phenomenex, injection volume: 5 or 10 µL, oven column temperature: 40 °C, flow rate: 0.15 mL/min, analysis duration: 70 min, PDA detection at wave lengths λ = 200–800 nm. The mobile phase consisted of (A) water with the addition of 5 mM HCOONH_4_ (pH 4.0) and (B) acetonitrile. The following gradient was applied: 0–1 min 0→3% B, 3–40 min 3→30% B, 40–50 min 30→95% B, 50–55 min 95% B, 55–57 min 95→3% B, equilibrium time—13 min in 3% B.

Conditions for the mass spectrometer were as follows: polarity positive: mass range *m*/*z* 100–800 Da, ion accumulation: 10 ms, interface temperature: 220 °C, heat block temperature: 220 °C, nebulizing gas flow: 1.5 L/min, drying gas pressure: 100 kPa, IS: +4.5 kV.

The calibration mixture was used to calibrate the TOF detector of the LCMS-IT-TOF mass spectrometer. The sample was prepared by dissolving 5 mM of buffer HCOONH_4_ in 1:1 ratio in the mixture of ACN and spinning in a centrifuge, and the supernatant was transferred to an HPLC injection vial.

### 4.6. Statistical Analysis

Data are expressed as mean values ± S.E.M. Multiple comparisons were analyzed using Kruskal-Wallis one-way analysis of variance by ranks. The statistical analyses were performed using STATISTICA StatSoft (Poland). Statistical significance was accepted at *p* < 0.05.

## Figures and Tables

**Table 1:** *Huperzia selago* gametophyte alkaloids identified by high resolution mass spectrometry and Formula Predictor software.

An alkaloid Name	Formula	Theoretical Mass	Measured Mass	*m*/*z* Error (ppm)
Fawcettimine	C_16_H_25_NO_2_	264.1958	264.1962	1.51
Deacetylfawcettine	C_16_H_27_NO_2_	266.2115	266.2109	−2.25
6*β*-hydroxyhuperzine A	C_15_H_18_N_2_O_2_	259.1441	259.1431	−3.86
16-hydroxyhuperzine B	C_16_H_21_N_2_O_2_	273.1598	273.1599	0.37
Deacetyllycoclavine	C_16_H_27_NO_2_	266.2115	266.2109	−2.25
Huperzine B	C_16_H_20_N_2_O	257.1648	257.1642	−2.33
Huperzine A	C_15_H_18_N_2_O	243.1492	243.1481	−4.52
Serratinidine	C_18_H_28_N_2_O_2_	305.2224	305.2217	−2.29
Annopodine	C_17_H_25_NO_3_	292.1907	292.1901	−2.05
Lycopodine	C_16_H_25_NO	248.2009	248.2002	2.82
Selagoline	C_16_H_25_NO	248.2009	248.1998	−4.48
Lycopecurine	C_16_H_27_NO	250.2165	250.2156	−3.60
Des-*N*-methylfastigiatine	C_18_H_26_N_2_O	287.2118	287.2113	−1.74
Flabelline	C_18_H_29_N_2_O	289.2274	289.2275	0.35
